# Effect of air pollution on adult chronic diseases: Evidence from a quasi-natural experiment in China

**DOI:** 10.3389/fpubh.2022.1105965

**Published:** 2023-01-13

**Authors:** Yan Li, Sheng Xu, Jinghua Yin, Guan Huang

**Affiliations:** ^1^College of Management, Guangdong AIB Polytechnic, Guangzhou, China; ^2^School of Health Management, Southern Medical University, Guangzhou, China; ^3^School of Insurance, Guangdong University of Finance, Guangzhou, China; ^4^Department of Economics, Jinan University, Guangzhou, China; ^5^Wenlan School of Business, Zhongnan University of Economics and Law, Wuhan, China

**Keywords:** air pollution, chronic diseases, winter heating policy, quasi-natural experiment, regression discontinuity design

## Abstract

We utilize a quasi-experiment derived from China's Huai River policy to investigate the effect of air pollution on adult chronic diseases. The policy led to higher pollution exposure in cities north of the river boundary because they received centralized coal-based heating supply from the government during winter, whereas cities in the south did not. By applying a geographic regression discontinuity design based on distance from the Huai River, we determine that a 10 μg/m^3^ increase in fine particulate matter (PM_2.5_) raises chronic diseases rates by 3.2% in adults, particularly cardiorespiratory system diseases. Furthermore, the same effects are observed on multiple chronic disease rates, but the rates are reduced to 1.3%. The effect of pollution exposure varies depending on age, gender, and urban/rural status. Our findings imply that reducing 10 μg/m^3^ of the average nationwide level of PM_2.5_ concentration will save 27.46 billion CNY (4.16 billion USD) in chronic disease costs.

## 1. Introduction

The prevention and treatment costs of chronic noncommunicable diseases account for the majority of public health expenditure. The last decades have seen an increase in the fraction of chronic noncommunicable disease prevention and treatment expenditure in China's medical expenditure. In 2003, the economic burden of chronic noncommunicable diseases in China was only 858.05 billion Chinese yuan (CNY) (130.01 billion USD),^1^accounting for 71.45% of the total economic burden of all diseases (1). By 2015, the economic cost of chronic noncommunicable diseases has reached 3682.8 billion CNY^2^(558 billion USD), accounting for 80% of the total economic burden of all diseases. In response to this, numerous attempts have been made to determine the causes of chronic diseases, including medical conditions, habits, genetics, and the environment (2, 3). Some studies have focused on the effect of air pollution on chronic diseases (4), However, most studies are limited to exploring the determinants of a single disease, making aggregating the total effect of air pollution on chronic diseases under the same framework difficult due to differences in context, methodologies, and pollutant measures across studies. Moreover, the causal relationship between air pollution and adult chronic diseases remains largely unexplored.

In the past few decades, as a rapidly growing developing economy, China's air quality has deteriorated to a certain extent with the increase of industrial pollution emissions. Simultaneously, as a large population country, China has the largest number of chronic patients in the world, and the prevalence of adult chronic diseases is rising with economic development. Therefore, China provides a unique opportunity to study the relationship between air pollution and adult chronic diseases. In the present work, we investigate in particular the causal relationship between air pollution and adult chronic diseases.

This study contributes to two strands of the literature. First, we build upon previous work that estimates the effect of air pollution on health. In contrast with a large number of studies that focused on mortality (5–8), migration (9), labor supply (10), body weight (11, 12), mental health (13), and children health (4, 14, 15), our study extends this work to encompass adult chronic diseases. Given the lack of research in this area, the total cost of air pollution is underestimated. Additional evidence from the cost of chronic diseases due to air pollution will be beneficial to policy makers and researchers to accurately calculate the total benefits of environmental regulation. Second, chronic diseases have considerably increased public health expenditure. Many previous studies have focused on complex and diverse determinants, including income (16), obesity (17), early life environment (18), genetics (2), and medical conditions (3). This study builds upon the growing literature by providing a new determinant of interest, namely, air pollution.

The primary challenge in identifying causal effect is that air pollution is likely to be endogenous due to omitted variable bias. For example, confusion factors related to economic activities, such as income, are among the determinants of health, while economic activities are also highly correlated with air pollution. To overcome the endogeneity of air pollution, the current study utilizes the regression discontinuity (RD) approach based on a quasi-natural experiment of China's Huai River policy. This policy creates discontinuous variation in air pollution by providing a coal-based centralized heating infrastructure only in cities north of the Huai River and no equivalent system for cities to the river's south.

Using the Huai River RD design, we find a statistically significant and economically positive effect of fine particulate matter (PM_2.5_) on chronic diseases. In particular, a 10 μg/m^3^ increase in average PM_2.5_ concentrations increases the rates of chronic diseases by 3.2% among adults. Taking advantage of a rich survey questionnaire, we define multiple chronic disease dummy variables when respondents answered two chronic diseases. We determine that a 10 μg/m^3^ (19.7%) increase in average PM_2.5_ concentrations increases the probability of having multiple chronic diseases by 1.3%. Moreover, these effects are nearly significant throughout an adult's life cycle, although the magnitude is different. In addition, these effects vary across urban–rural status and gender interaction groups, depending on the level of air pollution exposure. Our results are robust to varying specifications, including different controls, functional forms for the RD polynomial, bandwidth selection, sample selection, and placebo checks.

The Huai River effects are economically meaningful. Our estimate indicates that a 10 μg/m^3^ increase in PM_2.5_ concentrations induces a total annual cost of 27.46 billion CNY (4.16 billion USD), or 0.234% of China's gross domestic product (GDP), in terms of additional medical expenditure for chronic diseases. Notably, these values were derived from 2003 data. As GDP rises, the medical expenditure for chronic diseases in China increases.^3^ Several studies have found that the social mortality cost of a 10 μg/m^3^ increase in PM_2.5_ concentration is 48 billion USD (316.8 billion CNY) (7), the cost of obesity and being overweight is 18.9 billion CNY (2.86 billion USD) (11), the cost of mental illnesses is 12.68 billion USD (83.68 billion CNY) (13), and the total cost of medical expenditures is 75 billion CNY (11.36 billion USD) (19). Although the difference between the cost of chronic diseases due to air pollution and the cost of obesity due to air pollution estimated by Deschenes et al. (11) is the closest, directly comparing them is meaningless because our calculation is derived from China's data in 2003, while the values calculated by Deschenes et al. (11) are based on 2016 data. As China's economy grew rapidly from 2003 to 2016, a prediction can be made that if we use 2016 data to calculate the cost of chronic diseases, then the cost of chronic diseases caused by air pollution will be considerably greater than the cost of obesity. However, disregarding the effect of chronic diseases will certainly underestimate the overall health cost of air pollution.

The remainder of this paper is organized as follows. Section 2 presents the literature review and background information regarding the Huai River policy, and lays out the RD design. Section 3 describes the data sources. Section 4 presents our major findings, including those of the validity, robustness, and heterogeneous tests. Finally, Section 5 concludes the study.

## 2. Literature review and empirical strategy

### 2.1. Literature review

Air pollution is a fundamental determinant affecting population health. A large body of literature shows links between air pollution and exacerbations of respiratory diseases, preterm birth, infant mortality, low birth weight, neurodevelopmental disorders, deficits in lung growth, and possibly the development of asthma (20). In view of this fact, the medical mechanism of air pollution effects has always been a research focus. Several studies explore the medical mechanism of air pollution effects and believe that air pollution effects can be mediated by oxidative stress, chronic inflammation, endocrine disorders, and genetic and epigenetic mechanisms throughout the life cycle (21). Another research focus is the health costs of air pollution. Recent studies also estimate the cost of air pollution on a variety of health outcomes, including cardiorespiratory mortality, obesity, mental health, respiratory symptoms, asthma exacerbations, and asthma hospitalizations (4–8, 11, 12, 14, 15). The challenge associated with estimating the health costs of air pollution is that air pollution is likely to be endogenous due to omitted variable bias. There are different ways to solve the endogenous of air pollution in the literature in studying the health outcomes of air pollution. In order to address the endogenous of air pollution on body weight, Deryugina et al. (7) use changes in local wind direction as an instrumental variable for air pollution to estimate the life-years lost due to pollution exposure. Thermal inversion is another useful instrumental variable for air pollution. The strategy to instrument for air pollution using thermal inversion was first proposed by Arceo et al. (5), to evaluate the influence of air pollution on infant mortality in Mexico City. Subsequently, the strategy was used to explore the impact of air pollution on children's respiratory health (15), adult obesity (11), and mental illness (13). Another way to deal with the endogenous of air pollution is to use the exogenous variation of air pollution generated by policies, such as China's Huai River policy, which leads to higher pollution exposure in cities north of the river boundary, because they get centralized coal-fired heating from the government in winter, while southern cities do not. Almond et al. (22) first exploit RD design based on Huai River policy and find that the Huai River policy led to higher total suspended particulate levels in the north. This identification strategy has been subsequently used to explore the effects of air pollution on mortality, life expectancy (6, 23), and willingness to pay for clean air (24) in China. Although various health costs of air pollution have been estimated in the literature, the overall chronic disease and multiple chronic disease costs of air pollution have not received much attention under the condition of fully solving endogenous air pollution. We then extend the previous literature by using the Huai River RD design to investigate the cost of chronic diseases caused by air pollution.

### 2.2. Background

China's winter centralized heating system began in 1958. However, the government only provides centralized heating to northern cities due to energy and financial constraints (22, 23). The north–south boundary of China roughly runs along the Huai River and the Qinling Mountains. The government uses this line because it is also the line where the average temperature in January is about 0°C; however, it is not used for other administrative purposes (8, 24). Cities to the north of the river boundary receive centralized heating from the government every winter. By contrast, no centralized heating is provided by the state to southern China. Most centralized heating systems in the north are coal-fired. The incomplete combustion of coal during the heat generation process will lead to the release of air pollutants, particularly particulate matter. Many studies have found that the Huai River policy has led to an increase in the total level of suspended particulate matter in the northern region (22, 23).

The Huai River policy divides northern and southern cities into treatment and control groups, respectively. Coal consumption enables us to compare the difference in air pollution concentration between the two groups. This scenario provides a quasi-natural experimental environment for researchers to estimate the effect of air pollution on health by using the discontinuity of air pollution caused by coal burning in the Huai River.

This approach provides us with a useful research strategy for two reasons. First, some factors that affect health, such as economic level, education level, and resource differences, will be controlled because the Huai River Line is a geographical boundary rather than an economic and administrative boundary. This strategy enables us to identify the causal effects of air pollution on health. Second, the difference in pollution caused by the policy has always existed since its implementation in 1958. The pollution effect captured by this approach may be extremely significant due to the long-term cumulative effect of air pollution.

### 2.3. Empirical Strategy

Considering the effects of economic factors on health and the correlation between air pollution and these economic confounding factors, endogeneity may be produced by omitted variables between air pollution and chronic diseases among adults. To address this problem, we utilize the Huai River RD design. We estimate the effects of winter heating on air pollution and health by using an RD design based on distance from the Huai River. We examine whether discontinuous changes exist in air quality and chronic diseases among adults at the Huai River boundary.

In particular, we first estimate the first stage of air pollution by using an RD design created by the Huai River heating policy following Equation (1):


(1) $$\begin{array}{l} {PM}_{c}\;=\;\varphi_{0}\;+\;\varphi_{1}{North}_{c}\;+\;f\left({distance}_{c}\right)\;+\;\varphi_{2}^{{}^{\prime }}X_{c}\;+\;\varepsilon_{c} \end{array}$$

Where *PM*_*c*_ indicates the *PM*_2.5_ concentration (μg/m^3^) in county *c*; and *North*_*c*_ is a dummy variable for the north that takes the value of 1 if the county is in the north of the Huai River and 0 otherwise. The key independent variable, i.e., the running variable, *distance*_*c*_ is the distance between county *c* and the Huai River. We use positive values of *distance*_*c*_ for distances north of the Huai River and negative values for distances south of the river. The function *f* is a polynomial in *distance*_*c*_ whose coefficients are estimated in the regression. *X*_*c*_ is a covariate, and it includes temperature, relative humidity, cumulative precipitation, and sunshine duration. A potential problem with the Huai River RD design is that the space boundary is extremely long from the west to the east of China. Therefore, factors not observed in the east and west dimensions may confuse RD estimates. To solve this problem, our covariates also include the longitude quartile bin, which flexibly controls the systematic differences between the eastern and western dimensions. ε_*c*_ is the error term.

Second, the Huai River RD design utilizes the discontinuity in air pollution caused by coal-fired heating to estimate the effect of air pollution on health. We estimate the reduced form of the RD design to examine whether a discontinuous change exists in chronic diseases among adults at the Huai River boundary following Equation (2):


(2) $$\begin{array}{l} {Health}_{ic}\;=\;\alpha_{0}\;+\;\alpha_{1}{North}_{ic}\;+\;f\left({distance}_{ic}\right)\;+\;\alpha_{2}^{{}^{\prime }}Z_{ic}\;+\;\varepsilon_{ic} \end{array}$$

Where *i* indicates observation; and *Health*_*ic*_ denotes health status measures, particularly the indicators for chronic diseases, multiple chronic diseases, and subcategories of chronic diseases. The chronic diseases indicator take the value of 1 if *i* suffers from at least one chronic disease and 0 otherwise. *North*_*ic*_ takes the value of 1 if *i* is located in county *c* in the north of the Huai River and 0 otherwise. *distance*_*ic*_ indicates the distance from county *c* where individual *i* lives to the Huai River. *Z*_*ic*_ is a vector of observed covariates that potentially affect health, including not only *X*_*c*_ but also demographic and health behavior characteristics. The coefficient of interest, α_1_, measures a discontinuous change in health at the Huai River boundary.

Finally, some counties may have less air pollution due to environmental protection measures, although they are located in the north of the Huai River. We reestimate using a fuzzy RD framework with a two-stage least squares (2SLS) regression specified by Equations (3) and (4).


(3) $$\begin{array}{lll} {PM}_{ic}\; & = & \;\varphi_{0}\;+\;\varphi_{1}{North}_{ic}+f\left({distance}_{ic}\right)\;+\;\varphi_{2}^{{}^{\prime }}X_{ic}+\varepsilon_{ic} \end{array}$$

(4) $$\begin{array}{lll} {Health}_{ic}\; & = & \;\beta_{0}\;+\;\beta_{1}{PM}_{ic}\;+\;f\left({distance}_{ic}\right)\;+\;\beta_{2}^{{}^{\prime }}Z_{ic}\;+\;\varepsilon_{ic} \end{array}$$

Equations (3), (4) are the first and second stages, respectively, in a 2SLS system of equations. *PM*_*ic*_ refers to the exposure average concentration of *PM*_2.5_ sustained by individual *i* residing in county *c*. The other variables are as described above. We use *North*_*ic*_ as the instrument variable (IV) for *PM*_*ic*_. We estimate the effect of *PM*_2.5_ on health by using the fuzzy RD approach. The parameter of interest is β_1_, which measures the effect of *PM*_2.5_ exposure on chronic diseases after controlling for available covariates.

## 3. Data sources

We obtain chronic disease data from the China Family Panel Studies (CFPS), which is a nationwide and comprehensive social tracking survey project. CFPS aims to reflect the changes in China's society, economy, population, education, and health by tracking and collecting data at the individual, family, and community levels. We utilize rich questions and answers on chronic diseases in the CFPS 2010 wave, which is a baseline survey^4^ that includes the interviews of 14,960 households and 42,590 individuals from 162 counties/districts in 25 provinces, representing 95% of the population in China. CFPS is conducted by the Social Science Research Institute of Peking University. It uses implicit stratification, multiple stages [county/district (six-digit code),^5^ village/community, and household], multiple levels, and probability sampling in proportion to population size.

CFPS has four advantages for our research. First, accurate information about the geographic location of the sample from the county is crucial for our identification strategy. CFPS documents the geographic location and interview date of all the respondents, enabling us to match the health characteristics of the respondents accurately with the external air pollution data. Second, for our research content, CFPS provides detailed information about chronic diseases through the questions and answers and classification codes in the health questionnaire. Third, the survey covers men and women of different ages in rural and urban areas of China, enabling us to conduct rich heterogeneity analysis. Fourth, the survey not only provides health information but also detailed information on socioeconomic and demographic characteristics, enabling us to control a wide range of covariates.

We use three types of health measures. The first is chronic diseases, which is obtained from the questionnaire question “Have you ever suffered from any chronic disease diagnosed by your doctor in the last 6 months?” The variable of chronic diseases is assigned to 1 if the respondent suffers from at least one chronic disease and 0 otherwise. The second is multiple chronic diseases, which is obtained from the questionnaire question “What are the two most important chronic diseases you have been diagnosed with by your doctor?^6^” The result is assigned to 1 if the respondent answers two chronic diseases and 0 otherwise. The third is the subcategories of chronic diseases, which are obtained from the names and classification codes of the chronic diseases answered by the respondents.

For the running variable and longitude covariate, we first use ArcGIS to obtain the longitude and latitude of 162 counties surveyed in CFPS from the map of China. Second, we make a distance variable based on the locations of the county and the Huai River. In particular, we use ArcGIS to measure the shortest distance from the county centroids to the nearest point on the Huai River.^7^

Local governments in China are strongly encouraged to reduce air pollution in China, and the central government uses air quality readings to assess the environmental performance of local governments, and thus, researchers are concerned that local governments may manipulate data. Previously, several studies have investigated China's air pollution data and found a suspicious pattern in the distribution of reported data^8^ (25, 26). Although previous studies have shown that the satellite-based aerosol optical depth (AOD) retrieval pollution data and ground-based monitoring station measures exhibit no statistical difference (9, 27, 28), AOD-based data have higher accuracy than ground-based pollution data, which are more easily affected by weather conditions, because the former has a certain correction function for pollution diffusion caused by changes in meteorological conditions (29). To eliminate errors and improve accuracy, our data on air pollution are from satellite-based AOD retrievals. We obtain the AOD data from the *PM*_2.5_ concentration^9^ calculated by the Atmospheric Composition Analysis Group of Dalhousie University through sensors and processed using ArcGIS software. Wang et al. (30) showed that high ambient PM_2.5_ concentration is considered closely related to China's huge primary energy consumption, particularly coal consumption.

The weather data are obtained from the Daily Data Set of China's Surface Climate Data on the China Meteorological Science Data Sharing Service Website, which releases the daily weather variables of more than 800 meteorological stations in China. We use the inverse distance weighting method to convert the weather data from stations to counties and select a radius of 200 km. Weather data include temperature, relative humidity, cumulative precipitation, and sunshine duration. This dataset has been used in previous studies (9, 11, 13).

Air pollution and weather data were collapsed at the county level average in 2010 to match the CFPS baseline survey and meet the requirements of the Huai River RD design for cross-sectional characteristics. We compile a dataset from three data sources: the CFPS 2010 wave, air pollution, and weather data. Demographic variables (e.g., age, gender, minority, urban/rural status, and income) and health behavior variables (i.e., whether the respondent smokes regularly, drinks heavily, and eats excessive amounts of red meat), are obtained from the CFPS 2010 wave.

## 4. Empirical results

### 4.1. Major results

We begin investigating the effectiveness of the Huai River RD design. An essential test is whether systematic differences exist in observable determinants at the Huai River boundary. The RD design's identifying assumption is that observable determinants change smoothly at the boundary. Table 1 provides the summary statistics of county-level and individual-level observable covariates and evidence for the validity of the RD design. Columns 1 and 2 report the sample mean and standard deviation for the north and south of the Huai River. Column 3 reports the mean difference between the north and the south along with the associated standard errors. Notably, this statistic shows a simple difference, which is not necessarily a discontinuous difference at the boundary. In Column 4, we report whether a discontinuous change occurs at the Huai River boundary by using local linear regression, our primary RD specification in the empirical analysis, to approximate the size of the discontinuity estimated for the covariates with the bandwidth selected using the common mean square error (MSE) method proposed by Calonico et al. (31), Calonico et al. (32), and Calonico et al. (33) with a triangular kernel and report the standard errors in brackets. We do not find a statistically significant discontinuity for a wide range of observable determinants at the river boundary, confirming the internal validity of our research design.

**Table 1:** Summary statistics of observables for the north and south of the Huai River.

**Variable**	**North**	**South**	**Differences in means**	**RD estimates (Nonparametric)**
	**(1)**	**(2)**	**(3)**	**(4)**
**Weather and longitude**				
Temperature (°C)	11.095 (3.701)	18.404 (2.766)	−7.309^***^ [0.512]	0.272 [0.318]
Relative humidity (%)	61.904 (6.607)	73.646 (5.097)	−11.742^***^ [0.925]	−2.146 [1.583]
Precipitation (mm)	664.104 (278.597)	1447.153 (466.230)	−783.048^***^ [60.713]	35.448 [103.188]
Sunshine duration (h)	2190.358 (300.363)	1520.847 (354.240)	669.511^***^ [51.722]	115.746 [278.569]
Longitude	115.506 (6.887)	113.721 (6.412)	1.785^*^ [1.045]	1.916 [4.548]
**Demographic and health behavior characteristics**				
Age	45.160 (16.146)	45.924 (16.698)	−0.764^***^ [0.180]	−1.211 [0.738]
Gender	0.481 (0.500)	0.489 (0.500)	−0.008 [0.005]	0.003 [0.020]
Minority	0.952 (0.214)	0.872 (0.334)	0.080^***^ [0.003]	−0.009 [0.008]
Urban/rural status	0.413 (0.492)	0.524 (0.499)	−0.112^***^ [0.005]	−0.017 [0.030]
Income (10,000 yuan)	0.777 (1.552)	1.184 (2.372)	−0.407^***^ [0.022]	−0.069 [0.072]
Smoking regularly	0.309 (0.462)	0.287 (0.452)	0.022^***^ [0.005]	0.004 [0.023]
Drinking regularly	0.048 (0.214)	0.044 (0.205)	0.004^*^ [0.002]	−0.002 [0.011]
Excessive red meat consumption	0.009 (0.096)	0.019 (0.137)	−0.010^***^ [0.001]	−0.004 [0.003]
Consumption of puffed/fried food	1.252 (0.984)	1.339 (1.335)	−0.088^***^ [0.013]	0.000 [0.048]

Weather measurements were calculated as a county's average reading in the CFPS survey year. Columns (1) and (2) report the mean values of the north–south samples of the boundary, and Column (3) reports the raw differences between the mean of the two samples by using *t*-test. The results in Column (4) are the nonparametric estimated discontinuity at the Huai River obtained using local linear regression and the bandwidth selected using the common MSE method with a triangular kernel. The optimal bandwidth is chosen separately for each variable. In Columns (1) and (2), standard deviations are reported in parentheses. In Columns (3) and (4), standard errors are reported in brackets. ^*^Significant at 10% level; ^**^Significant at 5% level; ^***^Significant at 1% level.

We then present the RD results on the effect of the Huai River policy. The RD method allows for a transparent graphical representation of the effects of interest. We start from this analysis and then present the parametric and nonparametric estimation results. Before we proceed to the formal regression analysis, we provide a graphical analysis of the first stage of the RD design based on the regression results of Equation (1) and the reduced form of the RD design based on the regression results of Equation (2).

**Figure 1:**
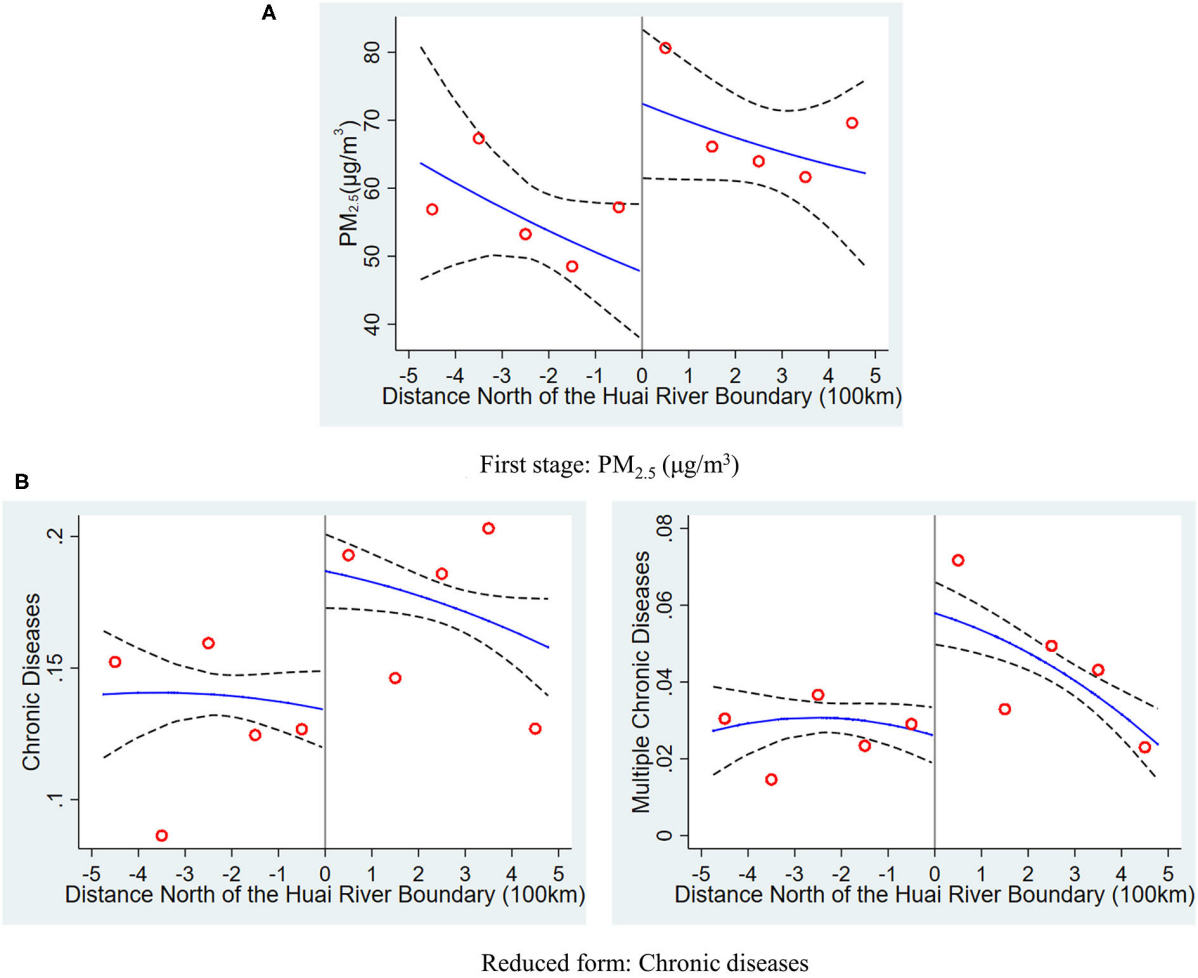
Distribution of pollution exposure and chronic diseases at the Huai river boundary. The graphs show the average value of PM_2.5_ exposure and rates of chronic diseases north and south of the Huai River. The horizontal axis is the distance north (positive values) and south (negative values) from the sample location to the Huai River. The scatterplot in **(A)** is the means of PM_2.5_ within 100 km bins, and the solid and dashed lines are the regression fit and associated confidence intervals, respectively, based on the quadratic polynomial regression of PM_2.5_ exposure on distance from the Huai River estimated separately on each side of the river. The right and left columns of **(B)** present the results for at least one chronic disease and multiple chronic diseases, respectively, estimated in the same manner as shown in **(A)**.

Figure 1A presents a graphical analysis of the first stage of the RD design. The points are the unconditional average values of *PM*_2.5_ across 100 km bins to the south and north of the Huai River boundary. The distance between counties and the boundary, *distance*_*c*_, is shown on the horizontal axis. The vertical line at *distance*_*c*_ = 0 indicates the location of the boundary. The northern counties are displayed on the right side of the vertical line, while the southern counties are presented on the left side. The solid and dashed lines are the regression fit and associated confidence intervals, respectively, based on the quadratic polynomial regression of *PM*_2.5_ exposure on the separately estimated distance from the Huai River. As evident from the figure, the discontinuity of *PM*_2.5_ concentration increases at the boundary, suggesting that the heating policy has caused higher pollution levels in the northern counties of the Huai River boundary. Similar findings have been reported in previous studies, such as Ebenstein et al. (6) and Ito and Zhang (24).

The right and left columns of Figure 1B display chronic disease and multiple chronic disease rates, respectively around the Huai River boundary, as estimated by the reduced form of the RD design. The figures indicate a discontinuous decrease in the adult chronic disease rate at the boundary, followed by a steady decrease. Visually, the discontinuous jumps in Figure 1B are around 6.9% and 4.8% for chronic diseases and multiple chronic diseases, respectively. These findings are consistent with the reduced form regression results presented in the next section. The figures depict an apparent dependency of the proportions of chronic disease and multiple chronic disease rates in adults on their location relative to the Huai River boundary. Although transparent graphics initially show the RD effect of interest, different regression methods and RD specifications, such as the choice of the order of the polynomial and the bandwidth, may exert a substantial effect on RD estimates.

In the major estimates, we use a polynomial *f* that is either linear or quadratic in *distance*_*i*_ by applying parametric and nonparametric regression methods. In the robustness checks, however, we also probe the sensitivity of the results to alternative RD specification.

Table 2 presents the first stage estimation results for PM_2.5_ when using parametric and nonparametric regression methods. The first two columns are the results when using a parametric method, while the last two columns are the results when using a nonparametric regression method. We report our estimates from local quadratic and linear regressions.^10^ The results in all regressions include weather and longitude covariates. The estimates are robust to different regression methods, the choice of polynomial for the running variable, and the inclusion of weather and longitude covariates. Table 2 suggests a discontinuous change in PM_2.5_ at the Huai River between 27 and 30 μg/m^3^. This magnitude is consistent with the visual evidence presented in Figure 1A. Following the work of Imbens and Lemieux (34) and Gelman and Imbens (35), we use the results from the local linear nonparametric regression application of the RD design as our baseline results.^11^ Column (4) presents our baseline results, which report the nonparametric estimates discontinuity at the Huai River by using the triangle kernel local linear regression and bandwidth selected by the common MSE-optimal bandwidth selector. Column (4) suggests a significant increase in PM_2.5_ at the Huai River. At the boundary, PM_2.5_ concentration rises by about 28.58 μg/m^3^.

**Table 2:** RD estimates of the effect of the Huai River policy on PM_2.5_.

	**Dependent variable: PM**_**2.5**_ **in** μ**g/m**^**3**^			

	**Parametric estimates**		**Nonparametric estimates**	
	**(1)**	**(2)**	**(3)**	**(4)**
North	29.917^**^ (12.983)	27.118^***^ (8.591)	27.094^**^ (11.489)	28.580^***^ (9.786)
Weather	Yes	Yes	Yes	Yes
Longitude	Yes	Yes	Yes	Yes
Polynomial	Quadratic	Linear	Quadratic	Linear
Size of bandwidth (100 km)	[−5 5]	[−5 5]	[−5.205 5.205]	[−4.026 4.026]
Observations	85	85	162	162
Observations inside bandwidth			88	78
Bandwidth selection method			MSE	MSE
Kernel			Triangle	Triangle

This table provides the parametric and nonparametric estimates of the effect of the Huai River Policy on PM_2.5_. In Columns (1) and (2), we report the parametric estimates of a dummy North = 1 for samples located to the north of the Huai River after controlling for quadratic and linear regressions in distance from the Huai River and its interaction with the dummy North, respectively. In Columns (3) and (4), we report the nonparametric estimate discontinuity at the Huai River by using triangle kernel local quadratic and linear regressions, respectively, and the bandwidth selected by the common MSE-optimal bandwidth selector. The results in all regressions include weather and longitude covariates. Standard errors are reported in parentheses. ^*^Significant at 10% level; ^**^Significant at 5% level; ^***^Significant at 1% level.

Table 3 provides the reduced-form and second-stage results for health outcomes when using parametric and nonparametric regression methods. Covariates from Table 1 were included in all regressions. Columns (1)–(4) report that the parametric regression approach and the sample locations are restricted to within 500 km of the Huai River. As mentioned earlier, this method can be regarded as an informal way to implement local regression by manually limiting bandwidth. Column (1) presents the reduced-form parametric estimation results when using a quadratic polynomial for *f*. These results are consistent with Figure 1B, and they provide evidence that a statistically significant discontinuous increase occurs in the rates of chronic diseases and multiple chronic diseases for adults at the Huai River boundary. Column (3) uses a linear polynomial. Columns (2) and (4) report the second-stage (2SLS IV) parametric estimation results. Nonparametric estimates from the reduced-form and second-stage (fuzzy RD) regressions are reported in Columns (5)–(8). Columns (5)–(8) provide the results when using triangle kernel local quadratic and linear regressions, respectively, and the bandwidth selected by the common MSE-optimal bandwidth selector.

**Table 3:** RD estimates of the effect of the Huai River policy and 10 μg/m^3^ of PM_2.5_ on health outcomes.

	**Parametric estimates**				**Nonparametric estimates**			
	**OLS**	**2SLS**	**OLS**	**2SLS**	**Reduced form**	**Fuzzy RD**	**Reduced form**	**Fuzzy RD**
**Dependent variables**	**(1)**	**(2)**	**(3)**	**(4)**	**(5)**	**(6)**	**(7)**	**(8)**
Chronic diseases	0.069^***^ (0.018)	0.021^***^ (0.006)	0.059^***^ (0.012)	0.019^***^ (0.004)	0.126^***^ (0.020)	0.044^***^ (0.007)	0.095^***^ (0.017)	0.032^***^ (0.006)
Multiple chronic diseases	0.048^***^ (0.010)	0.015^***^ (0.003)	0.027^***^ (0.007)	0.009^***^ (0.002)	0.068^***^ (0.012)	0.024^***^ (0.004)	0.061^***^ (0.011)	0.013^***^ (0.003)
**Subcategories of chronic diseases**								
Cardiorespiratory	0.055^***^ (0.012)	0.017^***^ (0.004)	0.035^***^ (0.008)	0.011^***^ (0.003)	0.080^***^ (0.012)	0.028^***^ (0.004)	0.064^***^ (0.011)	0.022^***^ (0.004)
Neuropsychiatric	0.011^**^ (0.005)	0.003^**^ (0.001)	0.006^*^ (0.003)	0.002^*^ (0.001)	0.019^***^ (0.006)	0.006^***^ (0.002)	0.012^***^ (0.004)	0.004^***^ (0.001)
Motor	0.024^***^ (0.009)	0.007^***^ (0.003)	0.018^***^ (0.006)	0.006^***^ (0.002)	0.060^***^ (0.011)	0.017^***^ (0.003)	0.039^***^ (0.009)	0.013^***^ (0.003)
Digestive	0.007 (0.009)	0.002 (0.003)	0.005 (0.007)	0.002 (0.002)	0.015 (0.010)	0.005 (0.003)	0.013 (0.009)	0.004 (0.003)
Other chronic diseases	−0.006 (0.010)	−0.002 (0.003)	0.006 (0.007)	0.002 (0.002)	−0.010 (0.011)	−0.003 (0.004)	−0.007 (0.010)	−0.002 (0.003)
Covariates	Yes	Yes	Yes	Yes	Yes	Yes	Yes	Yes
Polynomial	Quadratic	Quadratic	Linear	Linear	Quadratic	Quadratic	Linear	Linear
Size of Bandwidth (100 km)	[−5 5]	[−5 5]	[−5 5]	[−5 5]				
Observations	17752	17752	17752	17752	33575	33575	33575	33575
Kernel					Triangle	Triangle	Triangle	Triangle
Bandwidth selection method					MSE	MSE	MSE	MSE

Each cell in the table represents a separate regression. This table shows parametric [Columns (1)–(4)] and nonparametric [Columns (5)–(8)] estimates of the effect of the Huai River Policy and 10 μg/m^3^ of PM_2.5_ on the listed outcomes. Columns (1) and (3) report the ordinary least squares (OLS) estimates of the coefficient on a north of the Huai River dummy. Columns (2) and (4) present the 2SLS IV estimates by using the “North of Huai River” as the IV. Columns (1)–(2) and (3)–(4) show the results after controlling for quadratic and linear polynomials in distance from the Huai River interacted with a north dummy by using the sample within 500 km of the Huai River, respectively. Columns (5) and (7) report the estimated discontinuity at the Huai River. Columns (6) and (8) present the estimates of the effect of 10 μg/m^3^ of PM_2.5_ on the listed outcomes that regarded distance from the Huai River as the running variable and PM_2.5_ as the treatment variable, with the Huai River representing a “fuzzy” discontinuity at the level of 10 μg/m^3^ of PM_2.5_ exposure. Columns (5)–(6) and (7)–(8) show the results when using the triangle kernel local quadratic and linear regressions, respectively, and bandwidth selected by the common MSE-optimal bandwidth selector. Covariates from Table 1 were included in all regressions. Standard errors are reported in parentheses. ^*^Significant at 10% level; ^**^Significant at 5% level; ^***^Significant at 1% level.

Two characteristics of the results in Table 3 should be noted. First, the RD effects on chronic diseases and multiple chronic diseases are highly statistically significantly positive for different regression methods and RD specifications, implying that our conclusions are robust and not strongly affected by the choice of function form. Second, the coefficients estimated by the 2SLS IV method are smaller than the reduced-form estimates. Such finding is not surprising given that many counties have good air quality although they are located north of the Huai River because of regulatory measures. Therefore, the reduced-form estimate overvalues the effect of the Huai River policy.

As mentioned earlier, we use the results from the local linear nonparametric regression application of the RD design as our baseline results. The fuzzy RD approaches suggest a substantially smaller estimate of the health effects of PM_2.5_. Column (8), which reports the estimates from the nonparametric fuzzy RD approach, suggests that an additional 10 μg/m^3^ sustained exposure to PM_2.5_ is associated with a statistically significant increase in the probability of suffering from chronic diseases and multiple chronic diseases by 3.2 and 1.3%, respectively.

We also report the results of the change in subcategories of chronic diseases at the Huai River boundary. Our primary division is chronic diseases of the cardiorespiratory system, neuropsychiatric system, motor system, digestive system, and all other chronic diseases, which we have identified using the chronic disease classification codes recorded by CFPS and the Chinese coding scheme.^12^ We presume that chronic diseases of the cardiorespiratory system are the most affected by air pollution, while chronic diseases of the neuropsychiatric and motor systems are also partially affected by air pollution. Chronic diseases of the digestive system and other chronic diseases will not be affected by air pollution. This prediction is borne out by the data: a statistically significant increase in chronic diseases of the cardiorespiratory system, neuropsychiatric system, and motor system rates is found at the Huai River by using different RD specifications. The RD estimation coefficient for chronic diseases of the cardiorespiratory system is the largest. By contrast, the change in rate of all the other chronic diseases at the Huai River is a decrease at the Huai River line. However, this decrease is not statistically significant.

To make our conclusion more intuitive, we consider the following counterfactual policy. The policy changes coal-fired heating into natural gas, wind energy, or solar energy. Ma et al. (36) estimated that 15.5% of North China's PM_2.5_ emissions came from coal burning in power plants during winter.^13^ Assuming that coal-fired power generation in northern winter is used for heating.^14^ Therefore, if the output of existing coal burning power plants is replaced with wind power or solar power, PM_2.5_ will be reduced by 15.5%. This result implies a reduction in PM_2.5_ concentration by 7.86 μg/m^3^ for the average nationwide level of PM_2.5_ concentration in our data (50.72 μg/m^3^). The finding by Hu et al. (1) implied that China spends 858.054 billion CNY (130.01 billion USD) on treating chronic noncommunicable diseases every year. The application of the study's estimates suggests that the counterfactual policy will save 21.58 billion CNY (3.27 billion USD) in chronic disease costs. If the national average concentration of PM_2.5_ is reduced by 10 μg/m^3^, then the cost of chronic diseases will save 27.46 billion CNY (4.16 billion USD).

### 4.2. Robustness checks

We check the robustness and validity of our results against a variety of dimensions, such as functional forms for the RD polynomial, selection of bandwidth, sample selection, and placebo checks. Our robustness checks support the validity of the RD design, giving us confidence in the robustness of our results.

Although we control for the covariates listed in Table 1 in all regressions, one possible concern is that our RD estimates can be confused if covariates are discontinuous across the treatment threshold of the Huai River boundary. To address this concern, we examine the internal validity of our research design. If the variation near the Huai River boundary is real, then we expect that sample location characteristics, such as weather and longitude, and demographic and health behavior characteristics will not exhibit significant differences between the north and south of the Huai River. We use local linear regression and the bandwidth selected by the common MSE method proposed by Calonico et al. (31), Calonico et al. (32), and Calonico et al. (33) by using a triangular kernel, i.e., our major RD specification in the empirical analysis, to test for the continuity of sample characteristics at the Huai River boundary. The results are presented in Column (4) of Table 1 and the standard errors are in brackets. The RD estimates indicate that the discontinuous difference for the covariates is not statistically significant at the river boundary, confirming that observables that are close to the river boundary are identical on average.

In Table 4, we explore the robustness of the results to the selection of bandwidth to implement the parametric RD design by restricting the sample of locations, starting within 500 km of the Huai River in Column (1), which is primary analysis reported in Table 3, and then progressively narrowing the 100 km term to each subsequent selection of bandwidth as one moves from left to right. We report the results by using 2SLS IV estimates and controlling for a quadratic polynomial in distance from the Huai River and its interaction with a North dummy. The North dummy is the IV of PM_2.5_. Supplementary Table A1 further reports the first stage estimation for PM_2.5_. Although the results fluctuate to a certain extent, the discontinuity observed in chronic diseases, multiple chronic diseases, and subcategories of chronic diseases is significant at the level of 1%. This finding shows that our results are robust, even if we use samples that are closer to the Huai River.

**Table 4:** Effect of alternative bandwidths on regression results, parametric estimates.

	**IV Estimates of 10** μ**g/m**^**3**^ **of PM**_**2.5**_			

	**500 km**	**400 km**	**300 km**	**200 km**
**Dependent variables**	**(1)**	**(2)**	**(3)**	**(4)**
Chronic diseases	0.021^***^ (0.006)	0.039^***^ (0.007)	0.042^***^ (0.008)	0.026^***^ (0.005)
Multiple chronic diseases	0.015^***^ (0.003)	0.021^***^ (0.004)	0.024^***^ (0.005)	0.016^***^ (0.003)
**Subcategories of chronic diseases**				
Cardiorespiratory	0.017^***^ (0.004)	0.028^***^ (0.005)	0.028^***^ (0.005)	0.013^***^ (0.003)
Neuropsychiatric	0.003^**^ (0.001)	0.006^***^ (0.002)	0.007^***^ (0.002)	0.005^***^ (0.002)
Motor	0.007^***^ (0.003)	0.014^***^ (0.004)	0.020^***^ (0.004)	0.012^***^ (0.003)
Digestive	0.002 (0.003)	0.006^*^ (0.004)	0.006 (0.004)	0.006^**^ (0.003)
Other chronic diseases	−0.002 (0.003)	−0.003 (0.004)	−0.005 (0.005)	−0.003 (0.003)
Covariates	Yes	Yes	Yes	Yes
Observations	17,752	15,910	12,956	6,566

This table shows the 2SLS IV estimates of PM_2.5_ on the listed outcomes with the alternative size of bandwidth by using “North of Huai River” as IV after controlling for a quadratic polynomial in distance from the Huai River and its interaction with a North dummy. Column (1) presents the baseline regressions, which are the same as those reported in Column (2) of Table 3. The covariates from Table 1 are included in all regressions. Standard errors are reported in parentheses. ^*^Significant at 10% level; ^**^Significant at 5% level; ^***^Significant at 1% level.

Table 5 further explores the sensitivity of the nonparametric results to different bandwidth selection and kernel weighting methods. Column (1) reports the baseline regressions, and they are the same as those reported in Column (8) of Table 3. In the baseline regression, we use the triangle kernel local linear regressions and the bandwidth selected by the common MSE-optimal bandwidth selector. In Columns (2) and (3), we reestimate our MSE-optimal bandwidth results by using different kernel types (Epanechnikov and Uniform). To cross-validate the sensitivity of the results to different bandwidths, we use the same kernel local linear regressions as those in Columns (1)–(3) in Columns (4)–(6), but with an alternative bandwidth choice criterion. The common coverage error rate (CER) optimal bandwidth method proposed by Calonico et al. (32), Calonico et al. (37), and Calonico et al. (38) is used to estimate the effect of 10 μg/m^3^ of PM_2.5_ on health outcomes. The results are qualitatively similar across different bandwidth selection methods and choice of kernel type, suggesting that our findings are insensitive to the method used to generate our local linear regression estimates.

**Table 5:** Effects of alternative bandwidth selection and kernel weighting methods on regression results, nonparametric estimates.

	**Bandwidth: MSE-optimal bandwidth**			**Bandwidth: CER-optimal bandwidth**		
**Dependent variables**	**(1)**	**(2)**	**(3)**	**(4)**	**(5)**	**(6)**
Chronic disease	0.032^***^ (0.006)	0.031^***^ (0.006)	0.026^***^ (0.006)	0.023^***^ (0.005)	0.026^***^ (0.005)	0.020^***^ (0.005)
Multiple chronic diseases	0.020^***^ (0.004)	0.017^***^ (0.003)	0.013^***^ (0.003)	0.014^***^ (0.004)	0.079^***^ (0.040)	0.018^***^ (0.004)
**Subcategories of chronic diseases**						
Cardiorespiratory	0.022^***^ (0.004)	0.022^***^ (0.004)	0.010^***^ (0.002)	0.016^***^ (0.003)	0.016^***^ (0.003)	0.019^***^ (0.004)
Neuropsychiatric	0.004^***^ (0.001)	0.004^***^ (0.001)	0.002^*^ (0.001)	0.005^***^ (0.002)	0.004^***^ (0.002)	0.004^***^ (0.001)
Motor	0.013^***^ (0.003)	0.012^***^ (0.003)	0.007^***^ (0.003)	0.012^***^ (0.003)	0.012^***^ (0.003)	0.014^***^ (0.003)
Digestive	0.004 (0.003)	0.004 (0.003)	0.000 (0.002)	0.003 (0.003)	0.003 (0.003)	0.007^*^ (0.003)
Other chronic diseases	−0.002 (0.003)	−0.002 (0.003)	0.001 (0.002)	−0.005 (0.003)	−0.005 (0.003)	−0.001 (0.003)
Covariates	Yes	Yes	Yes	Yes	Yes	Yes
Observations	33575	33575	33575	33575	33575	33575
Kernel	Triangle	Epanechnikov	Uniform	Triangle	Epanechnikov	Uniform

This table shows the nonparametric estimates of the effect of PM_2.5_ on the listed outcomes with the Huai River representing a “fuzzy” discontinuity in the level of PM_2.5_ by using local linear regression different bandwidth selection and kernel weighting methods. Column (1) reports the baseline regressions, which are the same as those reported in Column (8) of Table 3. The table reports the bandwidth selection methods, i.e., one common MSE-optimal bandwidth selector or one common CER-optimal bandwidth selector. Covariates from Table 1 are included in all regressions. Standard errors are presented in parentheses. ^*^Significant at 10% level; ^**^Significant at 5% level; ^***^Significant at 1% level.

Overall, our parametric and nonparametric estimates for different bandwidths are qualitatively similar to those in our primary analysis and suggest that our core finding is insensitive to our bandwidth choice. Notably, the stability of the results in Tables 4, 5 further indicates that our major results in this study do not depend on parametric or nonparametric estimation methods.

In Table 6, we also report additional robustness checks for alternative specifications and sample the first stage estimation. We reproduce the analysis in Column (8) of Table 3 baseline estimates, in which we use the triangle kernel local linear regressions and the bandwidth selected by the common MSE-optimal bandwidth selector. We first examine the OLS estimates. In contrast with the equation estimated by OLS in Columns (1) and (3) of Table 3, Columns (2) and (3) in Table 6 report the traditional OLS equation that does not include a running variable for the distance from the Huai River and its interaction with the North dummy. Column (2) uses the full sample, while the sample within 500 km of the Huai River is used in Column (3). The traditional OLS estimates are remarkably smaller in magnitude compared with the baseline estimates of the health effects of PM_2.5_. In particular, the OLS estimates show that the coefficient of PM_2.5_ is between 0 and 0.003, and only the effect on chronic diseases is significant when using all samples. These estimates by conventional criteria can be explained by several reasons. One explanation is that under the non-RD design, the air pollution effect on health will be confused by economic factors, such as income, because air pollution exhibits a strong correlation with economic factors, which are also important determinants of health. Another primary explanation is the measurement error of air pollution. We cannot observe the precise air pollution exposure of each person, and thus, we can only use outdoor air pollution constructed from satellite observations to approximate actual personal pollution exposure. Many studies in the literature on the effect of air pollution have pointed out that allocating air pollution exposure at the regional level (the county level in the current study) to individuals will introduce classical measurement errors (5, 39–41). In turn, classical measurement errors will lead to the underestimation and confusion of air pollution effects.

**Table 6:** Robustness of results to alternative specifications and samples.

	**Baseline**	**OLS**	**OLS within 500 km**	**No Covariates**	**Hukou**	**Prefecture level**
	**(1)**	**(2)**	**(3)**	**(4)**	**(5)**	**(6)**
Chronic diseases	0.032^***^ (0.006)	0.003^***^ (0.001)	0.001 (0.001)	0.035^***^ (0.006)	0.035^***^ (0.006)	0.033^***^ (0.006)
Size of bandwidth (100 km)	[−2.749 2.749]			[−1.838 1.838]	[−2.415 2.415]	[−2.749 2.749]
Observations inside Bandwidth	12,044			5,927	10,582	12,044
Multiple chronic diseases	0.020^***^ (0.004)	0.001 (0.001)	0.000 (0.001)	0.019^***^ (0.004)	0.020^***^ (0.004)	0.021^***^ (0.004)
Size of bandwidth (100 km)	[−2.236 2.236]			[−2.656 2.656]	[−2.268 2.268]	[−2.236 2.236]
Observations inside bandwidth	10,217			12,044	9,859	10,217
Observations	33,575	33,575	17,752	33,575	30,898	33,575

This table presents the results of various robustness checks for alternative specifications and samples. Column (1) reports the baseline regressions, which are the same as those reported in Column (8) of Table 3. In Columns (3) and (4)–(6), we estimate the effect of PM_2.5_ on the listed outcomes with the Huai River representing a “fuzzy” discontinuity at the level of PM_2.5_ with a triangle kernel local linear regression and bandwidth selected by the common MSE-optimal bandwidth selector. Column (4) excludes weather, longitude, and demographic covariates. Column (5) excludes samples without local hukou registration. Column (6) collapses pollution data at the prefecture level, which typically includes 5 to 15 counties. In Columns (2) and (3), we report the OLS estimates of the association between PM_2.5_ and the listed outcome. All the results report the effect of 10 μg/m^3^ of PM_2.5_ on these outcomes. Standard errors are presented in parentheses. ^*^Significant at 10% level; ^**^Significant at 5% level; ^***^Significant at 1% level.

Our baseline results include covariates for weather and longitude variables in flexible specifications to control for the confusion of weather and longitude location factors on our results and ensure that the Huai River policy affects health through air pollution rather than weather and location differences. In Column (4), we exclude weather and longitude variables and find that the magnitude and statistical significance of the estimation coefficient are essentially unchanged.

As mentioned earlier, we construct the individual sustained pollution exposure variable based on the PM_2.5_ concentration in the residential county. A potential concern is that relocation to other counties may be required to work or seek cleaner air. Such migration, if it occurs, can pose a potential challenge to our pollution exposure measurement, which assumes that the level of exposure to pollution is at the level observed at their hukou (obtained at one's city of birth). In addition, large-scale migration will also pose potential challenges to the RD design based on the Huai River boundary if migration occurs across boundaries. Although some studies have pointed out that migration is unlikely to affect our estimates significantly due to strict migration policies and low real migration rates across boundaries (6, 24), we still explore the potential effect of migration on the results by using two methods. First, we exclude samples without local hukou registration because data collection on migrant populations is notoriously difficult. The results are presented in Column (5), and they remain robust. Second, job-oriented migration has a considerable rate of mobility within a prefecture-level city, which typically contains 5–15 counties. That is, people may live in one county but work in another. We collapse the pollution data at the prefecture level for RD estimation. The results are presented in Column (6), and they remain robust. Supplementary Table A2 further reports the same robustness checks for cardiorespiratory and non-cardiorespiratory chronic diseases. The results fail to contradict the study's qualitative findings.

The asymptotic properties of parametric and nonparametric estimators depend on the order of the polynomial and the bandwidth, respectively. A trade-off exists between the bias and variance of estimates: higher-order polynomials and smaller bandwidths reduce bias but increase variance. Here, we explore the sensitivity of our results to higher-order polynomials.

**Figure 2:**
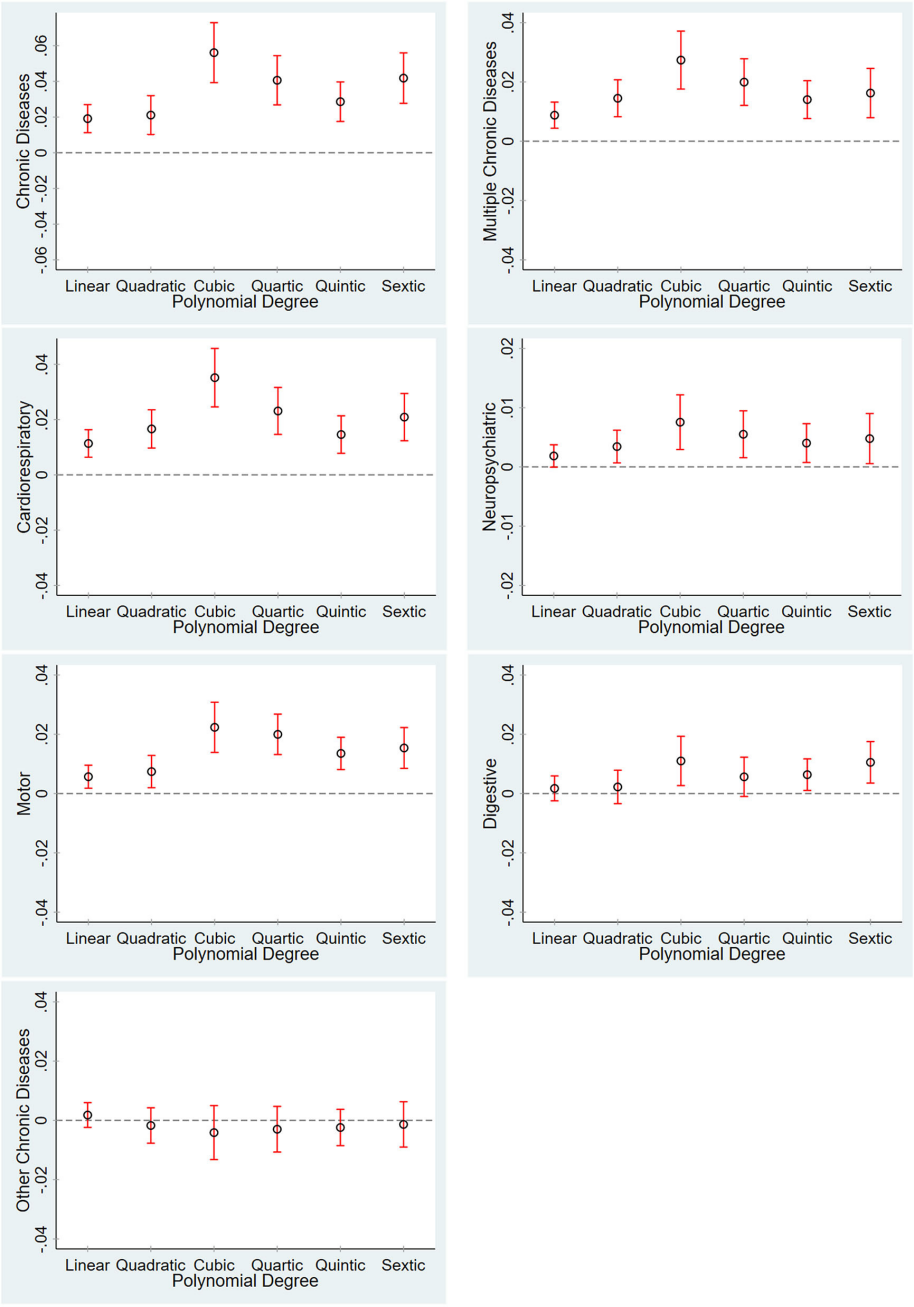
Robustness test for different RD polynomials. The graphs show the point estimates for the effect of PM_2.5_ on health outcomes and the associated 95% confidence intervals when the degree of the polynomial used in regression varies between linear and sextic. All the graphs show the 2SLS IV estimates and include all the covariates listed in Table 1.

Figure 2 plots the parametric estimates for the 2SLS effects of PM_2.5_ on health outcomes by using the sample of locations within 500 km of the Huai River and the associated 95% confidence intervals when the order of the polynomial varies between linear and sextic. The RD-estimated effects on chronic diseases, multiple chronic diseases, and subcategories of chronic diseases, including cardiorespiratory, neurological, and motor systems, are nearly always significantly positive. Our results are robust to the choice of functional forms for the RD polynomial.

The strong correlation between the Huai River Policy and chronic diseases in adults documented in Table 3 is unlikely to have arisen by chance. As a check on the model, we implemented a series of placebo tests. To do this, we used the RD nonparametric approach to estimate a reduced form boundary effect at a randomly assigned latitude boundary. To avoid having the placebo estimate influenced by any jump at the true boundary, we repeated the test 1,000 times, enabling us to exclude the possibility that our estimates are driven to a significant extent by small sample bias within groups. We use a triangle kernel local linear regression and the bandwidth selected by the common MSE-optimal bandwidth selector to estimate the effect of boundary on chronic and multiple chronic diseases. This test has been used in previous studies (42).

**Figure 3:**
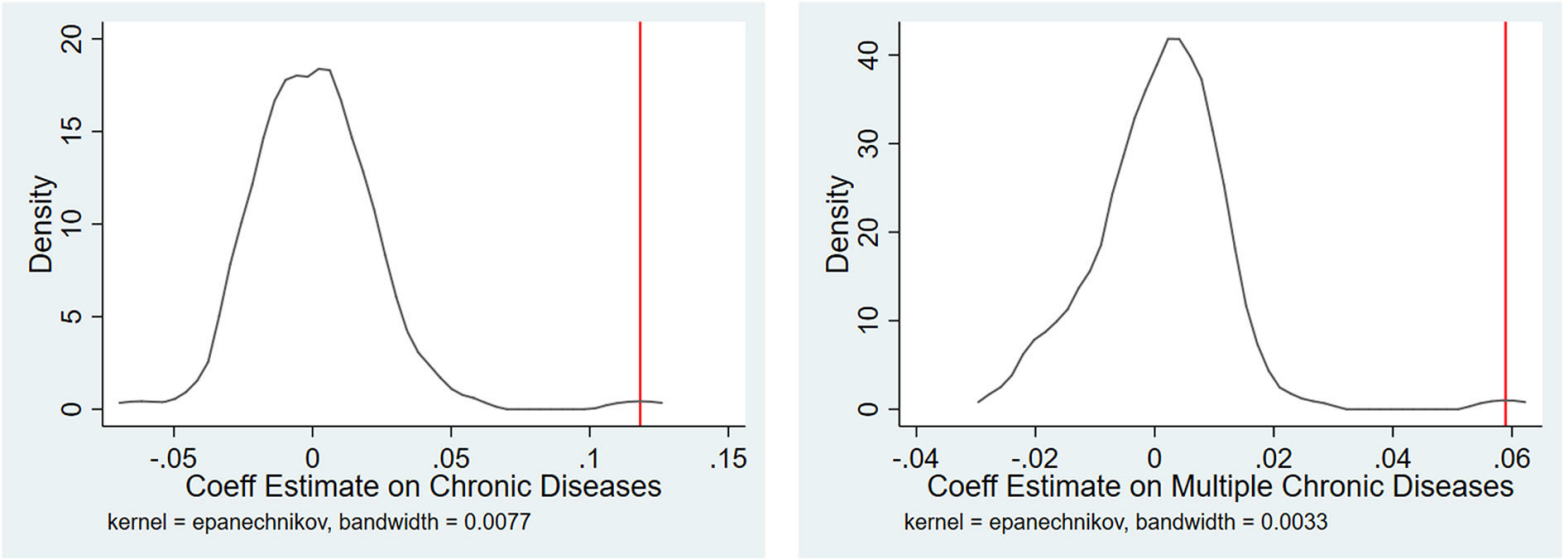
RD Estimates of the effect of the latitude boundary on chronic diseases, placebo estimates. The graphs show the distribution of the RD estimates by using a nonparametric method obtained from 1,000 random permutations of the boundary. Each placebo estimate assigned a false latitude boundary and then used a triangle kernel local linear regression and the bandwidth selected by the common MSE-optimal bandwidth selector to estimate the effect of the boundary on chronic and multiple chronic diseases. The vertical solid lines denote the actual estimates.

Figure 3 plots the distribution of placebo estimates along with the true discontinuity value for chronic and multiple chronic diseases. Each placebo estimate was obtained by assigning a latitude boundary at random, computing a “false” running variable as the distance from the sample location to the placebo boundary. The distributions of the placebos are centered at 0, and the actual coefficient estimates are plotted as vertical lines. As the graphs become clear, the true boundary effect (the Huai River Policy) on chronic and multiple chronic diseases is less than that of most of the placebo estimates. These findings exclude the possibility that the baseline estimates only averaged a small sample bias. They indicate that the odds of finding the Huai River boundary effects to be as large as ours merely by chance are small, and thus, our major results are not likely to be systematic artifacts caused by spurious factors around the Huai River boundary.

### 4.3. Heterogeneous effects

In the previous sections, we estimated the average effect of the Huai River policy on chronic diseases in adults. However, this effect can be heterogeneous. In this section, we investigate whether heterogeneity effects of the Huai River policy exist on chronic diseases. In particular, we explore heterogeneous effects on age, gender, and urban/rural registration.

We first examine how air pollution effect differs between young and elderly populations. The resistance of people varies with age, and the resistance of the elderly is generally weaker than that of the young; accordingly, multiple chronic diseases are more common among the elderly (43). Therefore, differences may exist in the effect of air pollution on chronic diseases among age groups. The wide age span of the CFPS data enables us to consider the effects of air pollution throughout adulthood. Table 7 presents the parametric and nonparametric estimates of PM_2.5_ exposure on chronic and multiple chronic disease rates separately for different age groups. The results of the subcategories of chronic diseases are reported in Supplementary Figure A1. As reported in Table 7, all the regressions include weather, longitude, and demographics other than age controls or covariates. We report the 2SLS IV estimates by using all the samples and the sample within 500 km of the Huai River and fuzzy RD estimates with a triangle kernel local linear regression. Our results show that the Huai River has significantly increased the prevalence of chronic diseases in adults aged 20–50 years. For multiple chronic diseases, the estimated coefficients are nearly significant throughout the adult life cycle, and magnitude increases with age. These results show that air pollution will not aggravate the prevalence of chronic diseases among the elderly, but will aggravate the incidence rate of multiple chronic diseases among the elderly. One explanation is that chronic diseases are extremely common among the elderly, and air pollution only aggravates the rate of suffering from multiple chronic diseases. For young people, regardless of whether chronic or multiple chronic diseases, air pollution has a significant positive effect on them, suggesting that PM_2.5_ is an important determinant of health. Supplementary Figure A1 plots the change in subcategories of chronic disease rates at the Huai River throughout the adult life cycle by using the local linear regression estimates of the magnitude of discontinuity, including the 95% confidence interval. The results indicate that the increase in cardiorespiratory chronic diseases is statistically significant for a large range of the adult life cycle. These results help explain the study's central finding of a large effect of PM_2.5_ on chronic diseases.

**Table 7:** Effect of the Huai River policy on chronic diseases by age.

	**Chronic diseases**			**Multiple chronic diseases**		

	**2SLS**		**Fuzzy RD**	**2SLS**		**Fuzzy RD**
**Age (years)**	**(1)**	**(2)**	**(3)**	**(4)**	**(5)**	**(6)**
<20	0.011 (0.009)	0.005 (0.015)	0.014 (0.014)	−0.001 (0.004)	−0.000 (0.001)	−0.002 (0.002)
[20, 30)	0.012^**^ (0.005)	0.027^***^ (0.008)	0.027^***^ (0.009)	0.002 (0.003)	0.007^*^ (0.004)	0.006^*^ (0.003)
[30, 40)	0.015^*^ (0.007)	0.019^**^ (0.009)	0.027^***^ (0.009)	0.008^**^ (0.003)	0.011^**^ (0.005)	0.011^***^ (0.004)
[40, 50)	0.031^***^ (0.008)	0.043^***^ (0.012)	0.052^***^ (0.012)	0.007^*^ (0.004)	0.010 (0.006)	0.013^*^ (0.007)
[50, 60)	0.012 (0.009)	0.022 (0.014)	0.027^**^ (0.014)	0.010^**^ (0.005)	0.016^**^ (0.008)	0.017^**^ (0.008)
[60, 70)	0.012 (0.012)	−0.002 (0.019)	0.014 (0.017)	0.020^***^ (0.007)	0.028^**^ (0.012)	0.031^**^ (0.012)
[70, 80)	0.021 (0.017)	0.018 (0.023)	0.042^*^ (0.023)	0.026^**^ (0.011)	0.029^*^ (0.016)	0.052^***^ (0.016)
≥80	−0.004 (0.030)	0.024 (0.042)	0.075^*^ (0.038)	0.023 (0.017)	0.027 (0.027)	0.046 (0.028)
Covariates	Yes	Yes	Yes	Yes	Yes	Yes
Polynomial	Quadratic	Quadratic	Linear	Quadratic	Quadratic	Linear
Size of bandwidth (100 km)	All	[−5 5]		All	[−5 5]	
Kernel			Triangle			Triangle
Bandwidth selection method			MSE			MSE

Each cell in the table represents a separate regression. In Columns (1)–(2) and (4)–(5), we report the 2SLS IV estimate of PM_2.5_ on the listed outcomes by using “North of Huai River” as the IV, after controlling for a quadratic polynomial in distance from the Huai River and its interaction with a North dummy. In Columns (3) and (6), we estimate the effect of PM_2.5_ on the listed outcomes with the Huai River representing a “fuzzy” discontinuity at the level of PM_2.5_ with a triangle kernel local linear regression, respectively, and the bandwidth selected by the common MSE-optimal bandwidth selector. Covariates from Table 1 other than age are included in all regressions. Standard errors are presented in parentheses. ^*^Significant at 10% level; ^**^Significant at 5% level; ^***^Significant at 1% level.

Second, we examined gender heterogeneity. Similar to Table 7, we report the parametric and nonparametric results and all the regressions, including weather, longitude, and demographics other than the age controls or covariates in Table 8. As indicated in Table 8, when estimated separately for men and women, evidence of consistencies across genders in the effect of PM_2.5_ on chronic diseases and multiple chronic diseases seems to be strong. For example, in the preferred specifications for using local linear regression in Columns (3) and (6), we estimate that the rate of men and women with chronic diseases at the Huai River increased by 3.3%, respectively. Similarly, the rate of men and women with cardiorespiratory chronic diseases at the Huai River increased by 2.1 and 2.3%, respectively. The rate of men and women with motor chronic diseases at the Huai River increased by 1.4 and 1.1%, respectively. The results between different genders are similar in quality, which is consistent with the interpretation of the results driven by joint exposure to air pollution, rather than a false correlation with omitted variables.

**Table 8:** Heterogeneous effect of the Huai river policy on chronic diseases by gender.

	**Men Only**			**Women Only**		
	**2SLS**		**Fuzzy RD**	**2SLS**		**Fuzzy RD**
	**(1)**	**(2)**	**(3)**	**(4)**	**(5)**	**(6)**
Chronic diseases	0.016^***^ (0.005)	0.020^***^ (0.008)	0.033^***^ (0.008)	0.015^***^ (0.005)	0.021^***^ (0.008)	0.033^***^ (0.008)
Multiple chronic diseases	0.009^***^ (0.003)	0.009^**^ (0.004)	0.014^***^ (0.004)	0.011^***^ (0.003)	0.019^***^ (0.005)	0.023^***^ (0.005)
**Subcategories of chronic diseases**						
Cardiorespiratory	0.011^***^ (0.003)	0.015^***^ (0.005)	0.021^***^ (0.005)	0.009^***^ (0.003)	0.018^***^ (0.005)	0.023^***^ (0.005)
Neuropsychiatric	0.001 (0.001)	0.005^**^ (0.002)	0.006^***^ (0.002)	0.002 (0.001)	0.002 (0.002)	0.002 (0.002)
Motor	0.006^**^ (0.002)	0.006 (0.004)	0.014^***^ (0.004)	0.007^**^ (0.003)	0.009^**^ (0.004)	0.011^***^ (0.004)
Digestive	0.000 (0.003)	0.001 (0.004)	0.002 (0.004)	0.001 (0.003)	0.003 (0.004)	0.006 (0.005)
Other chronic diseases	0.002 (0.003)	−0.001 (0.004)	−0.002 (0.004)	−0.001 (0.003)	−0.003 (0.005)	−0.003 (0.005)
Covariates	Yes	Yes	Yes	Yes	Yes	Yes
Polynomial	Quadratic	Quadratic	Linear	Quadratic	Quadratic	Linear
Size of Bandwidth (100 km)	All	[−5 5]		All	[−5 5]	
Kernel			Triangle			Triangle
Bandwidth selection method			MSE			MSE

This table replicates the RD estimates in Table 7, but estimated by gender. Covariates from Table 1, other than gender, are included in all regressions. Standard errors are presented in parentheses. ^*^Significant at 10% level; ^**^Significant at 5% level; ^***^Significant at 1% level.

Finally, regional heterogeneity may exist for two reasons. First, China's rural residents are considerably poorer than its urban residents. Given that income levels play an important role in food intake and the incidence of chronic diseases (44), urban residents may have a high prevalence of chronic diseases, confusing the effects of air pollution. We expect that the effect of air pollution on chronic diseases is insignificant in urban areas. Second, pollution in cities is more serious due to industrial agglomeration (45), and thus, the total air pollution exposure of rural residents may be significantly lower than that of urban residents. We expect that the effect of air pollution in urban areas will be greater.

Table 9 provides the 2SLS IV and local linear estimates of PM_2.5_ on chronic and multiple chronic diseases by urban/rural-specific. We analyze our preferred specification by using local linear regression results reported in Columns (3) and (6). For chronic diseases, the effect of PM_2.5_ is statistically significant in rural areas but not in urban areas. The different effects may be related to the income gap between urban and rural areas. Given the higher income level of urban residents and their excessive calorie intake, urban residents have a higher rate of chronic disease. This income-driven chronic disease effect may be sufficient to offset the effect of air pollution. Consequently, the estimated coefficient of urban samples is insignificant. For multiple chronic diseases, the estimation coefficient is significantly positive across urban and rural areas. However, compared with rural area residents, urban area residents have a higher risk of multiple chronic diseases during adulthood. This comparison supports the effect of total exposure to air pollution on chronic diseases and the argument that cities are more exposed to air pollution.

**Table 9:** Heterogeneous effect of the Huai river policy on chronic diseases by urban/rural status.

	**Urban only**			**Rural only**		
	**2SLS**		**Fuzzy RD**	**2SLS**		**Fuzzy RD**
	**(1)**	**(2)**	**(3)**	**(4)**	**(5)**	**(6)**
Chronic diseases	0.026^***^ (0.005)	0.051^***^ (0.010)	0.004 (0.010)	0.012^***^ (0.006)	0.005 (0.007)	0.028^***^ (0.007)
Multiple chronic diseases	0.017^***^ (0.003)	0.028^***^ (0.006)	0.034^***^ (0.007)	0.006^*^ (0.003)	0.009^**^ (0.004)	0.012^**^ (0.005)
**Subcategories of chronic diseases**						
Cardiorespiratory	0.010^***^ (0.004)	0.031^***^ (0.007)	0.036^***^ (0.008)	0.012^***^ (0.003)	0.010^**^ (0.005)	0.008^**^ (0.004)
Neuropsychiatric	0.002^*^ (0.001)	0.003 (0.002)	0.004^**^ (0.002)	0.001 (0.001)	0.004^*^ (0.002)	0.006^**^ (0.002)
Motor	0.010^***^ (0.003)	0.015^***^ (0.005)	0.018^***^ (0.004)	0.005^*^ (0.003)	0.004 (0.004)	0.010^***^ (0.004)
Digestive	0.002 (0.003)	0.007 (0.005)	0.008 (0.005)	−0.001 (0.003)	−0.001 (0.004)	−0.001 (0.003)
Other chronic diseases	0.006^**^ (0.003)	0.010^*^ (0.006)	0.007 (0.007)	−0.003 (0.003)	−0.008^*^ (0.004)	−0.009^**^ (0.004)
Covariates	Yes	Yes	Yes	Yes	Yes	Yes
Polynomial	Quadratic	Quadratic	Linear	Quadratic	Quadratic	Linear
Size of bandwidth (100 km)	All	[−5 5]		All	[−5 5]	
Kernel			Triangle			Triangle
Bandwidth selection method			MSE			MSE

The results report the discontinuity in the listed outcomes by urban/rural status in the same manner as those reported in Tables 7, 8. Covariates from Table 1, other than urban/rural status, are included in all regressions. Standard errors are presented in parentheses. ^*^Significant at 10% level; ^**^Significant at 5% level; ^***^Significant at 1% level.

**Figure 4:**
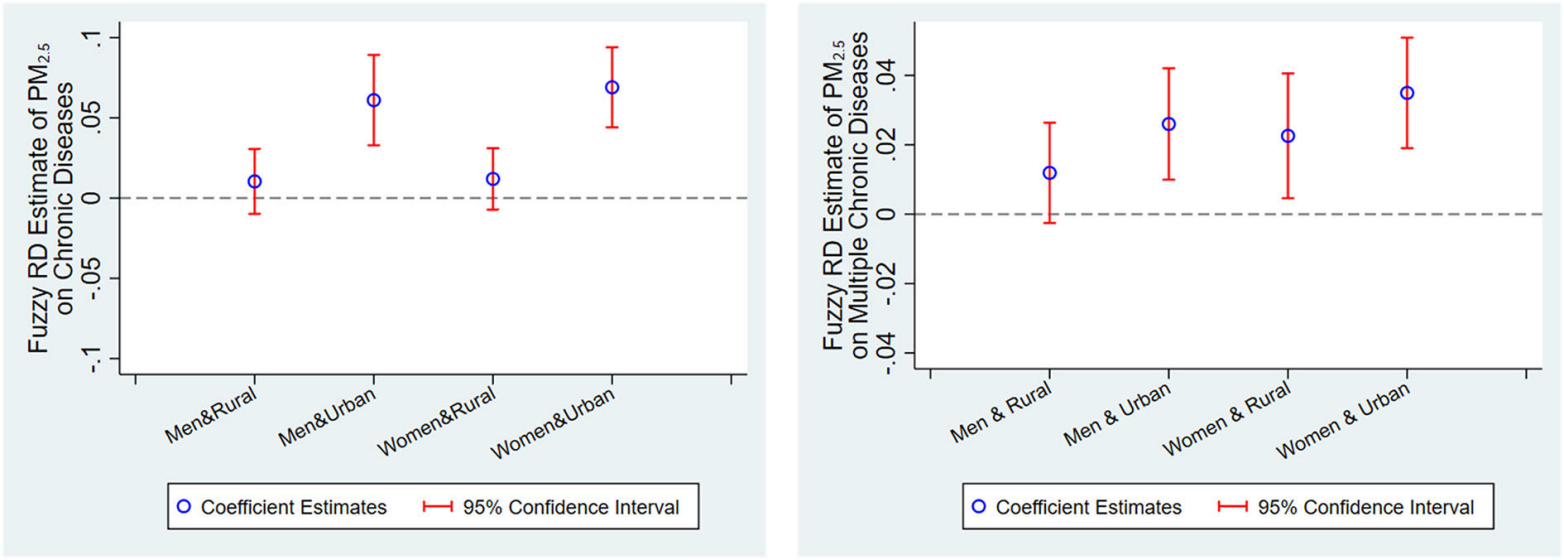
Heterogeneous effect of additional 10 μg/m^3^ exposure to PM_2.5_ on chronic diseases by gender and urban/rural status. These graphs present fuzzy RD nonparametric point estimates of the effect of additional 10 μg/m^3^ exposure to PM_2.5_ on chronic and multiple chronic diseases, regarding distance from the Huai River as the running variable and PM_2.5_ as the treatment variable, with the Huai River representing a “fuzzy” discontinuity at the level of pollution exposure by gender and urban/rural status and the associated 95% confidence intervals. The discontinuities are estimated using a triangle kernel local linear regression and the optimal bandwidth is chosen by the common MSE-optimal bandwidth selector. Each point estimate includes all the covariates, other than gender and urban/rural status, listed in Table 1.

Although we do not find evident gender heterogeneity from Table 8, gender heterogeneity may be confused by urban–rural differences. For example, differences exist in the amount of physical labor between men and women in rural China, but it may not exist in cities. More manual work (physical activities) can reduce the incidence of many diseases (46, 47), leading to gender heterogeneity being different between rural and urban samples. To test this hypothesis, we further divide the rural and urban samples into four subsamples based on gender and estimate the effect of the Huai River policy on the rural male, rural female, urban male, and urban female populations. As reported in the graph on the right of Figure 4, no significant gender difference is found in the effect of the Huai River policy on multiple chronic diseases in urban samples, but differences are observed in rural samples. In particular, the effect of PM_2.5_ on rural women is statistically significant, but the effect on rural men is insignificant. This comparison supports the conjecture that differences exist in the amount of physical labor between rural men and women. In addition, gender difference in rural samples may also be explained by the difference in total exposure to air pollution between men and women. Women are mostly engaged in outdoor agricultural production (e.g., work on the field), while men are mostly engaged in non-agricultural work in rural China. Given the nature of their work, the total exposure to air pollution of rural women may be significantly higher than that of rural men. The same results are found for cardiorespiratory chronic diseases, as presented in the graph on the left of Supplementary Figure A2.

### 4.4. Mechanism tests

Two main conclusions can be drawn from the results obtained so far. First, air pollution increases the risk of chronic diseases in adults. Second, in addition to cardiorespiratory system diseases, a statistically significant increase in chronic diseases of the neuropsychiatric system and motor system rates is also found at the Huai River by using different RD specifications. Many studies show that air pollution has a direct impact on cardiorespiratory diseases (6, 23). In this section, we mainly explore the possible channels of air pollution affecting the neuropsychiatric system and motor system diseases. First, air pollution may affect neuropsychiatric system diseases such as mental health directly through the induction of systemic or brain-based oxidative stress and inflammation. The biological pathway of fine particulate matter inducing oxidative stress and inflammation is that they will lead to the disorder of cytokine signal transduction, which can lead to depression, anxiety, cognitive dysfunction, and other mental illness (48). Second, air pollution may also affect neuropsychiatric system diseases through sleep disorders, which can lead to depression and anxiety. For motor system diseases, air pollution may affect it through behavioral responses. Many studies find that people are likely to stay indoors to avoid air pollution, thereby increasing sedentary behaviors and reducing outdoor physical activities (49), which causes diseases of the motor system. We explore differences in sleep time and physical activity frequency to explain the effects of air pollution on chronic diseases of the neuropsychiatric system and motor system. To gain some insights into these factors, we use rich questions and answers about exercise and sleep in the CFPS. Like the above specification, we report the results of parametric and nonparametric estimates.

Table 10 presents the fuzzy RD estimates of PM_2.5_ exposure on the frequency of physical activity and sleep time of adults. We start with sleep time in Table 10. We find that higher concentrations of air pollution can lead to a reduction in sleep time, and when using our preferred specifications, Column (3), this effect is significant. As mentioned earlier, Column (3) uses the optimal bandwidth method, while Columns (1), (2) show insignificant positive results due to manual bandwidth restriction. We then examine the effect of PM_2.5_ on physical activity. The survey asked how often an individual engages in fitness or participates in physical activity, ranging from 1 (1 time in a few months) to 5 (almost daily). Since only 6,140 observations report a valid range, these estimates should be interpreted with caution. Nevertheless, we find a stable negative effect of PM_2.5_ on physical activity frequency, indicating that less outdoor exercise to avoid air pollution is a possible channel for the air pollution effect.

**Table 10:** Potential mechanisms of the Huai River policy on chronic diseases.

	**2SLS**		**Fuzzy RD**

	**(1)**	**(2)**	**(3)**
Sleep time	0.026 (0.017)	0.020 (0.027)	−0.075^**^ (0.034)
Physical activity frequency	−0.024 (0.021)	−0.166^**^ (0.072)	−0.110^*^ (0.060)
Covariates	Yes	Yes	Yes
Polynomial	Quadratic	Quadratic	Linear
Size of bandwidth (100 km)	All	[−5 5]	
Kernel			Triangle
Bandwidth selection method			Mserd

Each cell in the table represents a separate regression. The dependent variables are and sleep time and physical activity frequency. Physical activity frequency is expressed in terms of how often an individual engages in fitness or participates in physical activity, ranging from 1 (1 time in a few months) to 5 (almost daily). Sleep time is expressed as the number of hours of sleep on weekdays. Standard errors are presented in parentheses. ^*^Significant at 10% level; ^**^Significant at 5% level; ^***^Significant at 1% level.

## 5. Conclusions

Although the previous literature has examined the shorter lifespan caused by airborne particulate matter, limited evidence is available regarding the effect of air pollution on chronic diseases. We used an RD design based on distance from the Huai River to estimate the chronic disease consequences of the indoor heating policy. A fuzzy RD estimate finds that sustained exposure to additional 10 μg/m^3^ PM_2.5_ is associated with a reduction of 3.2 and 1.3% in at least one chronic disease and multiple chronic disease rates, respectively. Various robustness checks and placebo regressions using false latitude boundary support our findings. Our findings suggest that the effect of air pollution differs depending on urban or rural status, gender, and age. In particular, women who work outdoors in agricultural production in rural areas are more sensitive to air pollution. For age heterogeneity, the coefficient of estimation of multiple chronic diseases is nearly significant throughout the adult life cycle, and its size increases with age. For young people, regardless of whether they are suffering from chronic diseases or multiple chronic diseases, air pollution exerts a significant positive effect on them, indicating that PM_2.5_ is an important determinant of health.

The results of this study have powerful policy implications. We demonstrate that negative effect of air pollution on chronic diseases. Considering that the annual cost of chronic diseases in China is 858.054 billion CNY (130.01 billion USD), the estimates of this study imply that reducing 10 μg/m^3^ of the average nationwide level of PM_2.5_ concentration will save 27.46 billion CNY (4.16 billion USD) in chronic disease costs.

In addition, in pursuing economic development, developing countries are prone to disregard emission restrictions on pollutants, leading to the deterioration of air quality and the increase in the economic burden of diseases. Whether health risks caused by air quality reduce labor productivity and human capital, which, in turn, hinders economic development, further research is necessary. More broadly, the results of this study are of enlightening significance for environmental regulations, economic incentives, and labor policies.

## Data availability statement

Publicly available datasets were analyzed in this study. This data can be found at: http://www.isss.pku.edu.cn/cfps/.

## Author contributions

All authors listed have made a substantial, direct, and intellectual contribution to the work and approved it for publication.

## Footnotes

^1^For comparison, we use the annual average exchange rate between USD and CNY in 2010: 1 USD for 6.6 CNY in this study.

^2^See https://www.sohu.com/a/306526685_120099842 (in Chinese).

^3^Hu et al. (1) calculated the economic burden of chronic noncommunicable diseases in China in 2003. Health expenditure generally increases with GDP growth, and thus, our estimate is low bound.

^4^CFPS conducted four consecutive tracking interviews in 2012, 2014, 2016, and 2018. Our RD design uses the cross-section data of CFPS 2010.

^5^The three administrative levels, i.e., province, city, and county, are respectively marked as two-digit, four-digit, and six-digit codes in China. See http://www.stats.gov.cn/tjsj/tjbz/tjyqhdmhcxhfdm/.

^6^If the respondent has multiple chronic diseases, then the two most important diseases should be provided. If the respondent has only one chronic disease, then the answer to the second disease should be “not applicable”.

^7^This distance ranges from 2 km to 2200 km, and the mean distance is 560 km.

^8^Chen et al. (9) found that AOD-based pollution data in China are closely matched with data from ground-based monitoring stations.

^9^PM_2.5_ refers to particles with an equivalent aerodynamic diameter of 2.5 μm or less in ambient air.

^10^The first two columns of reports come from the RD method, which limits the samples to 500 km of Huai River. This method can be regarded as an informal approach for implementing local quadratic and linear methods by manually limiting bandwidth.

^11^Gelman and Imbens (35) suggested that the parametric RD methods have several undesirable statistical properties, and local linear regression based on data near RD cutoff may produce the most robust estimation.

^12^The codes are provided by the Chinese Center for Disease Control and Prevention.

^13^Ma et al. (36) developed an emission inventory for 2013 by using up-to-date information on energy consumption and emission controls. They simulated the contribution of coal combustion to the total concentration of PM_2.5_ in 74 major cities across the country. Notably, this study is about emissions from coal burning power plants in northern China during winter, not overall coal usage.

^14^Evidently, this situation is different from the reality, because most of the heating is directly generated by a coal-fired boiler in an apartment building. Building accurate emissions is frequently challenging, and thus, we would like to emphasize that our calculation should be interpreted as a rough estimation.
