# Supplementary material for: Effect of air pollution on adult chronic diseases: Evidence from a quasi-natural experiment in China
Source: Front Public Health. 2023 Jan 13;10:1105965. doi: 10.3389/fpubh.2022.1105965 (PMC9880427; doi:10.3389/fpubh.2022.1105965)
Supplement: Supplementary file 1 [file Data_Sheet_1.docx]

Supplementary Material

Effect of Air Pollution on Adult Chronic Diseases: Evidence from a Quasi-natural Experiment in China

Yan Li, Sheng Xu*, Jinghua Yin, and Guan Huang

*** Correspondence:** Sheng Xu: shenghsu@163.com

# Appendix A

**Table A1.** Effect of Alternative Bandwidths on Regression Results, First Stage Estimation

|  | Dependent Variables: 10 µg/m^3^ of PM_2.5_ | | | |
| --- | --- | --- | --- | --- |
|  | 500 km | 400 km | 300 km | 200 km |
|  | (1) | (2) | (3) | (4) |
| North | 3.295***  (0.075) | 2.781***  (0.081) | 2.731***  (0.077) | 4.935***  (0.086) |
| First-stage F-Stat | 1921.4 | 1172.33 | 1234.26 | 3275.44 |
| Covariates | Yes | Yes | Yes | Yes |
| Observations | 17752 | 15910 | 12956 | 6566 |
| Note: This table shows results for the first stage estimation in equation (3) with alternative choices of bandwidth, which corresponds to the 2SLS IV estimation in Table 4. The covariates from Table 1 are included in all regressions. Standard errors are reported in parentheses. * significant at 10% level; ** significant at 5% level; *** significant at 1% level. | | | | |

**Table A2.** Robustness of Additional Results for the Effect of the Huai River Policy on Subcategories of Chronic Diseases

|  | Baseline | OLS | OLS within 500 km | No Covariates | Hukou | Prefecture Level |
| --- | --- | --- | --- | --- | --- | --- |
|  | (1) | (2) | (3) | (4) | (5) | (6) |
| Cardiorespiratory | 0.022***  (0.004) | 0.002***  (0.001) | 0.003***  (0.001) | 0.021***  (0.004) | 0.022***  (0.004) | 0.023***  (0.004) |
| Size of Bandwidth (100 km) | [−2.835 2.835] |  |  | [−1.906 1.906] | [−2.547 2.547] | [−2.835 2.835] |
| Observations inside Bandwidth | 12773 |  |  | 6374 | 10704 | 12773 |
| Non-cardiorespiratory | 0.009*  (0.005) | 0.000  (0.001) | −0.001  (0.001) | 0.007  (0.005) | 0.011**  (0.004) | 0.009*  (0.005) |
| Size of Bandwidth (100 km) | [−3.167 3.167] |  |  | [−3.769 3.769] | [−2.940 2.940] | [−3.167 3.167] |
| Observations inside Bandwidth | 13644 |  |  | 15633 | 11814 | 13644 |
| Observations | 33575 | 33575 | 17752 | 33575 | 30898 | 33575 |
| Note: This table replicates the RD estimates in Table 6, but with dependent variables, i.e., cardiorespiratory and non-cardiorespiratory diseases. All the results report the effect of 10 µg/m^3^ of PM_2.5_ on these outcomes. Standard errors are presented in parentheses. * significant at 10% level; ** significant at 5% level; *** significant at 1% level. | | | | | | |


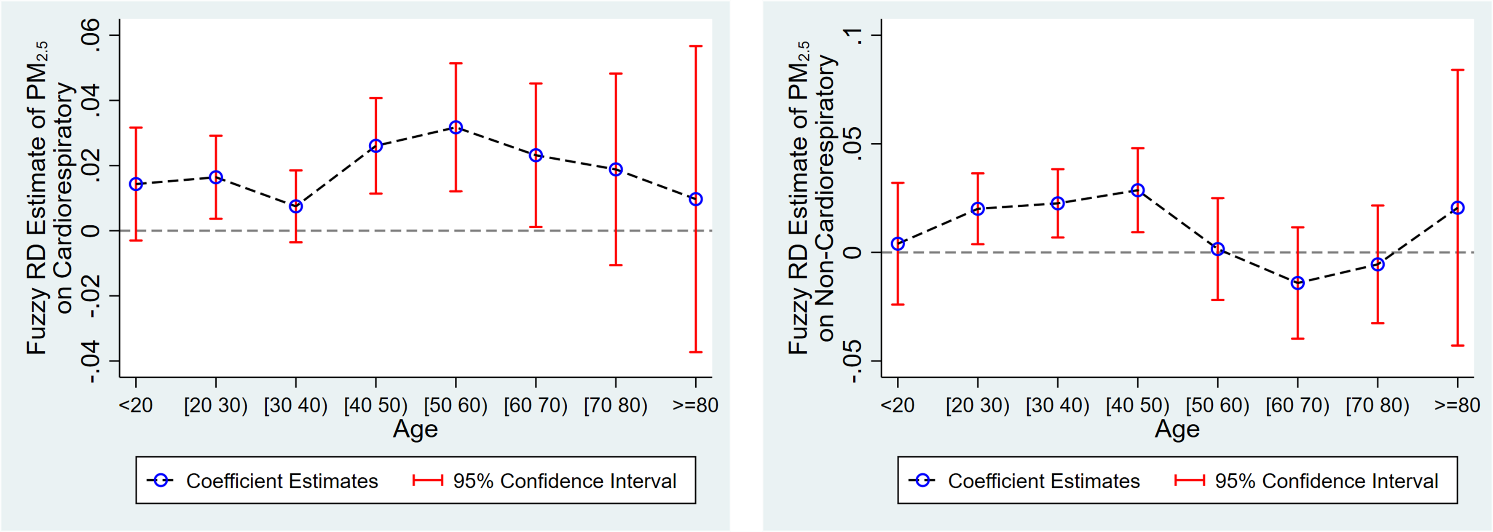


**Figure A1.** Effect of Additional 10 μg/m^3^ Exposure to PM_2.5_ on the Subcategories of Chronic Diseases by Age. Note: These graphs show fuzzy RD nonparametric point estimates of the effect of additional 10 μg/m^3^ exposure to PM_2.5_ on cardiorespiratory and non-cardiorespiratory diseases at 10-year age intervals and associated 95% confidence intervals, regarding distance from the Huai River as the running variable and PM_2.5_ as the treatment variable, with the Huai River representing a “fuzzy” discontinuity at the level of pollution exposure. The discontinuities are estimated using a triangle kernel local linear regression and the optimal bandwidth chosen by the common MSE-optimal bandwidth selector.


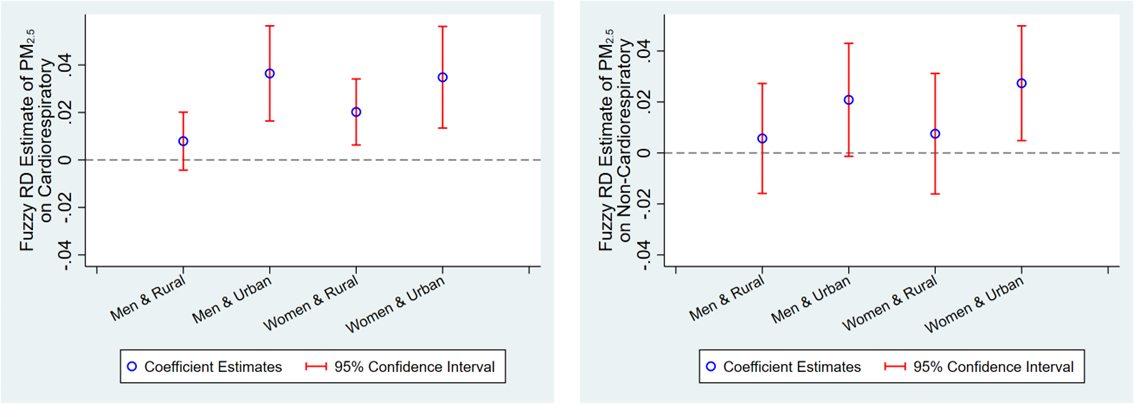


**Figure A2.** Effect of Additional 10 μg/m^3^ Exposure to PM_2.5_ on the Subcategories of Chronic Diseases by Gender and Urban/Rural Status. Note: These graphs show fuzzy RD nonparametric point estimates of the effect of additional 10 μg/m^3^ exposure to PM_2.5_ on cardiorespiratory and non-cardiorespiratory diseases, regarding distance from the Huai River as the running variable and PM_2.5_ as the treatment variable, with the Huai River representing a “fuzzy” discontinuity at the level of pollution exposure by gender and urban/rural status and the associated 95% confidence intervals. The discontinuities are estimated using a triangle kernel local linear regression and the optimal bandwidth chosen by the common MSE-optimal bandwidth selector.
